# Muscular Atrophy in Tabes Dorsalis

**Published:** 1889-12-07

**Authors:** 


### MUSCULAR ATROPHY IN TABES DORSALIS.

M. Déjerine, from an examination of twenty cases, in nine of which he obtained autopsies, comes to the conclusion that the wasting of the muscles—which, in his experience, occurs in 20 per cent. of cases of locomotor ataxy—is not an accident or complication, but an integral part of the pathological process. It comes on at a late period in the course of the disease and progresses slowly, is mostly symmetrical, and advances faster in the lower than in the upper extremities, leading in the former to talipes equinus with flexion of the toes, especially the great toe, and in the latter to the condition known as the Aran-Duchenne's paralysis, or more rarely scapulo-humeral or ante-brachial. These are purely paralytic, but in the foot contraction of the aponeuroses assists in producing the deformity. Its cause is progressive wasting of the motor nerves, which may be extremely attenuated to the very origin of the anterior roots. This atrophy is exclusively peripheral, the motor cells and the grey matter of the anterior cornua remaining unaffected. Voluntary contractility is diminished or lost, faradic and galvanic modified, but the reaction of degeneration is seldom observed.

